# Fungal Communities in Leaves and Roots of Healthy-Looking and Diseased *Ulmus glabra*

**DOI:** 10.3390/microorganisms10112228

**Published:** 2022-11-10

**Authors:** Adas Marčiulynas, Diana Marčiulynienė, Jūratė Lynikienė, Remigijus Bakys, Audrius Menkis

**Affiliations:** 1 Lithuanian Research Centre for Agriculture and Forestry, Institute of Forestry, Liepų Str. 1, Girionys, LT-53101 Kaunas, Lithuania; 2 Department of Forestry, Kaunas Forestry and Environmental Engineering University of Applied Sciences, Liepų Str. 1, Girionys, LT-53101 Kaunas, Lithuania; 3 Department of Forest Mycology and Plant Pathology, Uppsala BioCenter, Swedish University of Agricultural Sciences, P.O. Box 7026, SE-75007 Uppsala, Sweden

**Keywords:** *Ulmus glabra*, Dutch elm disease, biodiversity, climate change, tree health

## Abstract

The aim of this study was to investigate fungal communities associated with leaves and roots of healthy-looking and declining *U. glabra* trees. The study was expected to demonstrate whether and how the diversity and composition of fungal communities change in these functional tissues following the infection by Dutch elm disease-causing fungi. The study sites included six *U. glabra* sites in Lithuania, where leaves and roots were sampled. DNA was isolated from individual samples, amplified using ITS2 rRNA as a marker, and subjected to high-throughput sequencing. The sequence analysis showed the presence of 32,699 high-quality reads, which following clustering, were found to represent 520 non-singleton fungal taxa. In leaves, the fungal species richness was significantly higher in healthy-looking trees than in diseased ones (*p* < 0.05). In roots, a similar comparison showed that the difference was insignificant (*p* > 0.05). The most common fungi in all samples of roots were *Trichocladium griseum* (32.9%), *Penicillium restrictum* (21.2%), and Unidentified sp. 5238_7 (12.6%). The most common fungi in all samples of leaves were *Trichomerium* sp. 5238_8 (12.30%), *Aureobasidium pullulans* (12.03%), *Cladosporium* sp. 5238_5 (11.73%), and *Vishniacozyma carnescens* (9.86%). The results showed that the detected richness of fungal taxa was higher in samples collected from healthy-looking trees than from diseased ones, thereby highlighting the negative impact of the Dutch elm disease on the overall fungal diversity.

## 1. Introduction

Wych elm (*Ulmus glabra*) is an ecologically important tree species in the dendroflora of the northern Baltic Sea region [1]. In Lithuania, a significant part of these trees grows on riverbanks and slopes. Individual trees, their groups, or even smaller monocultures can be found at these sites. However, old-growth trees are very rare or missing due to the ongoing Dutch elm disease (DED), which has been reported in Lithuania [2]. Apart from the loss of valuable trees, DED threatens many *U. glabra*-associated organisms [3]. Prior to the emergence of DED in Europe, *Ulmus* spp. was associated with a large number of different organisms [4]. For example, 79 insect species were shown to be exclusively dependent on *Ulmus* spp. trees [5]. Moreover, 39 different epiphytic lichens were shown to be associated with *U. glabra*, including several endangered species [6]. Furthermore, endangered fungi such as *Rhodotus palmatus* or *Hymenochaete ulmicola* are also associated with *U. glabra* [7,8]. The latter demonstrates that *U. glabra* provides valuable habitats and is of key importance for biodiversity.

DED is one of the most devastating tree diseases in the northern hemisphere [9]. DED is a vascular wilt disease caused by pathogenic fungi of the genus *Ophiostoma* including less virulent *Ophiostoma ulmi* and the more virulent *O. novo-ulmi* and *O. himal-ulmi* [10]. Diseases that damage plant vascular systems, i.e., wilt diseases, cause significant damage, thereby, plants may become stunted, show wilt symptoms, and ultimately die [11]. There have been two pandemics in the last century. The first one started in the 1920s and was caused by *O. ulmi*, which over time killed up to 40% of *Ulmus* spp. trees in Europe. The second and ongoing pandemic started in the early 1970s and was caused by *O. novo-ulmi*, which has rapidly spread to different parts of Europe, killing more than one billion mature *Ulmus* spp. trees [9,12,13]. The disease is also common in North America, Central Asia, and Japan, i.e., in principle, it follows the distribution of host trees [14]. In Lithuania, DED has also severely devastated *Ulmus* spp. stands, resulting in a limited number of individuals remaining [2]. As DED continues to spread, it is likely that most mature *Ulmus* trees will be destroyed in infested areas [15]. *Ophiostoma* pathogenic fungi are propagated by elm bark beetles (*Scolytus* spp. and *Hylurgopinus* spp.) [16,17]. The larvae of these beetles overwinter under the bark of dying elm trees along with the pathogen. A new generation of beetles emerges in the spring and feeds on healthy elm trees. At the time of feeding, they spread conidia of the pathogen into the xylem of healthy trees and cause clogging and cavitation, resulting in foliage wilting and eventual tree death [18,19]. A typical internal symptom of this disease is the formation of a brown ring in the infected sapwood [20]. Irreversible processes begin in trees, and if the plant is able to survive for some time, it will still become less resistant to various other diseases and pests. Such damaged trees become a favorable environment for beetles to overwinter and reproduce [21]. DED can also be spread from tree to tree via root contacts of densely growing trees, which is very important given the preservation of still healthy trees [20].

Recent studies show that pathological conditions are an important factor in determining the structure of fungal communities in different tissues [22]. Such findings coincide with the fact that *Ulmus* trees with low susceptibility to disease have a less diverse fungal community in the xylem than highly susceptible trees [23]. However, recent research on DED has focused mostly on endophytes living in the rhizosphere, phyllosphere, and in internal tissues of leaves, roots, and stems, as they have been shown to play an important role in tree resistance against biotic and abiotic stress [24,25,26]. Meanwhile, there was less attention on how DED influences the diversity and composition of fungal communities in different tissue of *Ulmus* spp.

The aim of this study was to investigate fungal communities associated with the leaves and roots of healthy-looking and declining *U. glabra* trees. We hypothesized that fungal communities in different functional tissues change depending on the state of tree health and that competition between indigenous fungi and newly invading fungi may occur.

## 2. Materials and Methods

### 2.1. Study Sites and Sampling

**Figure 1:**
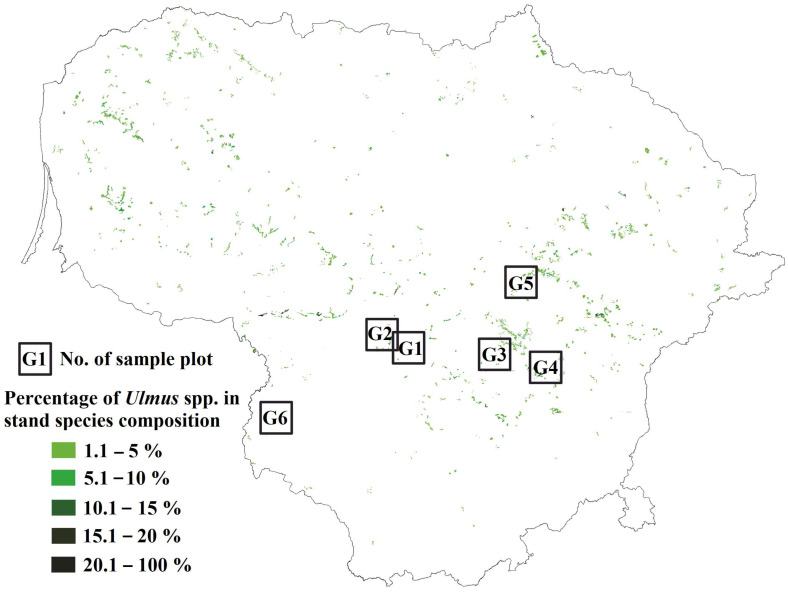
Map of Lithuania showing the distribution and composition of *Ulmus* spp. in stands (in green), where sampling of leaves and roots from healthy and diseased trees was carried out.

The study sites were in central Lithuania Figure 1. In Lithuania, *Ulmus* spp. dominated forest stands are absent, yet these tree species can be found in mixed broadleaved forest stands as an admixture species in the canopy or as a component of the understory. Such habitats are frequently found in forest stands of central Lithuania Figure 1. The study sites in Table 1 were broadleaved forest stands that were between 50 and 90-year-old and with an admixture of *Ulmus* spp. These were commonly located in relatively fertile soils of slopes and ravines near perennial water streams. The only exception was site G1, which was dominated by conifers in a canopy; but accommodated a rich understory of various broadleaved tree species, including elms. All sampled *Ulmus* trees were of young age (26–38-year-old), approximately 11–20 m in height and 10–15 cm at DBH, growing in shaded conditions in the understory. The characteristic symptoms of DED (trees with individual branches with wilting leaves, as well as individual dead branches) were observed on elms at each site.

Samples of leaves and roots in each site were taken from 12 healthy-looking and 12 declining trees. In each site, the minimum distance between healthy-looking and declining trees was ca. 50 m. Samples were taken in July 2019. Leaves were collected by cutting branches from the lower part of the crown (4–12 m from the ground) using telescopic secateurs. Individual leaf samples consisted of 10 leaves from different branches, which were collected using sterilized hand-held secateurs and placed in separate plastic bags. Instruments were carefully cleaned between individual samples. Samples of leaves, which were collected from healthy-looking trees, showed no symptoms of DED or other diseases. By contrast, samples of leaves, which were collected from declining elms branches, showed black or dark brown necroses on leaves, and tree branches were with clear internal symptoms of DED. A main lateral root (ca. 1 cm thick) with fine roots was excavated under each symptomatic and asymptomatic tree and placed in a separate plastic bag. Collected samples were transported the same day to the laboratory and stored at −20 °C.

### 2.2. DNA Isolation, Amplification, and Sequencing

The principles of DNA work followed the study by Marčiulynas et al. [29]. Prior to the isolation of the DNA, each sample (leaves and roots) was freeze-dried. Before freeze drying, the fine roots were washed twice with running distilled water and cut into smaller pieces. Leaf samples were not additionally sterilized. After the freeze-drying, ca. 0.03 g dry weight of each leaf or root sample was placed into a 2 mL screw-cap centrifugation tube together with glass beads. No surface sterilization of samples was carried out. Samples were homogenized using a Fast prep shaker (Bertin Technologies, Montigny-le-Bretonneux, France). The DNA was isolated using a CTAB extraction buffer (0.5 M EDTA pH 8.0, 1 M Tris-HCL pH 8.0, 5 M NaCl, 3% CTAB) [29]. Amplification of ITS2 rRNA region was performed using a fungal-specific primer gITS7 [30] and a universal primer ITS4 [31], both containing sample identification barcodes. Samples of the same tree and the same substrate were amplified using primers with the same barcode. PCR was performed in 50 μL reactions and consisted of the following final concentrations, 0.25 ng/μL—template DNA, 200 μM of dNTPs; 750 μM of MgCl_2_; 0.025 μM DreamTaq Green polymerase (5 U/μL) (Thermo Scientific, Waltham, MA, USA), and 200 nM of each primer. Amplifications were performed using the Applied Biosystems 2720 thermal cycler (Applied Biosystems, Foster City, CA, USA). The PCR program started with denaturation at 95 °C for 5 min, followed by 30 cycles of 95 °C for 30 s, annealing at 56 °C for 30 s and 72 °C for 30 s, followed by a final extension step at 72 °C for 7 min. The PCR products were assessed using gel electrophoresis on 1% agarose gel stained with Nancy-520 (Sigma-Aldrich, Stockholm, Sweden). PCR products were purified using 3 M sodium acetate (pH 5.2) (Applichem Gmbh, Darmstadt, Germany) and 96% ethanol mixture (1:2). After quantification of PCR products using a Qubit fluorometer 4.0 (Life Technologies, Singapore), samples were pooled in an equimolar mix and used for PacBio sequencing using one SMRT cell (SciLifeLab, Uppsala, Sweden).

### 2.3. Bioinformatics

The sequences generated were subjected to quality control and clustering in the SCATA NGS sequencing pipeline at http://scata.mykopat.slu.se (accessed on 10 August 2022). Quality filtering included the removal of short sequences (<200 bp), sequences with low read quality, primer dimers, and homopolymers, which were collapsed to 3 base pairs (bp) before clustering. Sequences that were missing a tag or primer were excluded. The primer and sample tags were then removed from the sequence, but information on the sequence associated with the sample was stored as meta-data. The sequences were then clustered into different taxa using single-linkage clustering based on 98% similarity. The most common genotype (real read) for each cluster was used to represent each taxon. For clusters containing two sequences, a consensus sequence was produced. Fungal taxa were taxonomically identified using GenBank (NCBI) database and the Blastn algorithm. The criteria used for identification were sequence coverage > 80%, and similarity to taxon level 98–100%, similarity to genus level 94–97%. Sequences not matching these criteria were considered unidentified and were given unique names. Representative sequences of fungal non-singletons are available from GenBank under accession numbers OP467028–OP467547.

### 2.4. Statistical Analyses

Rarefaction analysis was performed using Analytical Rarefaction v.1.3, available at http://www.uga.edu/strata/software/index.html (accessed on 15 August 2022). The Shannon diversity index and qualitative Sørensen similarity index were used to characterize the diversity and composition of fungal communities [32,33]. The nonparametric Mann-Whitney test in Minitab was used to test if the Shannon diversity index among different plots and samples was statistically similar or not. The composition of fungal communities was also studied using non-metric multidimensional scaling (NMDS) based on the Bray-Curtis similarity index. One-way ANOSIM was performed to test for significant differences among different substrates. Tukey’s method was used to create a set of confidence intervals between the means. These analyses were performed using Vegan 2.5.7 [34] and Stats 3.6.2 in R 4.1.1 (https://www.r-project.org accessed on 20 August 2022) [35].

## 3. Results

High-throughput sequencing generated 32,699 high-quality reads. Clustering analysis showed the presence of 520 non-singleton taxa. Table 2, while singletons were removed. Among fungal taxa in root samples, 147 (57.9%) were represented Ascomycota, 84 (33.1%)–Basidiomycota, 17 (6.7%)–Zygomycota, 3 (1.2%)–Glomeromycota, 2 (0.8%)–Chytridiomycota and 1 (0.3%)–Mucoromycota. In leaf samples, Ascomycota was represented by 205 (63.1%) fungal taxa, Basidiomycota–119 (36.6%), and Zygomycota–1 (0.3%) (Supplementary Tables S1 and S2. The number of high-quality sequences and fungal taxa from each study site is in Table 2. The Shannon diversity index of fungal communities ranged between 0.32 and 3.42 in the roots of DED-infected trees and between 0.94 and 2.90 in the roots of healthy-looking trees. In leaves, these values were between 3.09 and 3.20 and between 2.83 and 3.10, respectively Table 2. The Shannon diversity index did not differ significantly either between root samples of healthy-looking and diseased trees or between corresponding leaf samples (*p* > 0.05). However, it was significantly higher when compared between leaf and root samples from healthy-looking trees and between leaf and root samples from declining trees (*p* < 0.05). The Sørensen similarity index of fungal communities between root samples of diseased and healthy-looking trees of *U. glabra* was 0.24–0.37 (low), and between corresponding leaf samples, it was 0.50–0.59 (moderate).

**Figure 2:**
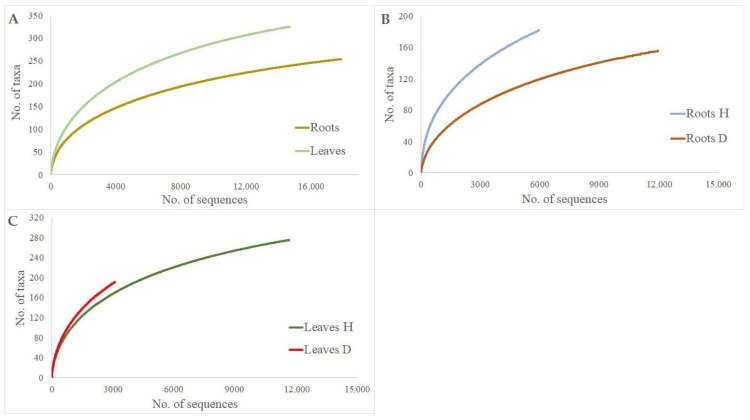
Species accumulation curves showing the relationship between the cumulative number of fungal taxa and the number of ITS rRNA sequences from *Ulmus glabra* root and leave samples: (**A**) total roots vs. total leaves; (**B**) roots of healthy trees (Roots H) vs. roots of diseased trees (Roots D); (**C**) leaves of healthy trees (Leaves H) vs. leaves of diseased trees (Leaves D).

The rarefaction analysis showed that a plot of fungal taxa vs. the number of fungal sequences resulted in curves, which did not reach the asymptote when all sites were taken together Figure 2. When the same number of sequences was taken, the species richness in the roots of healthy trees was significantly higher than in the roots of DED-infected ones (*p* < 0.05) Figure 2B. However, the richness of fungal taxa was similar in the damaged vs. undamaged leaves (*p* > 0.05). Nevertheless, the total number of sequences was ca. six times higher in healthy than in damaged leaves Figure 2C.

In leave and root samples from healthy-looking and diseased trees, the richness of fungal taxa varied among individual study sites Table 2. However, leaf samples from healthy-looking trees had a significantly higher fungal richness than those from DED-infected trees (*p* < 0.05), and a significant difference was not detected between corresponding root samples (*p* > 0.05).

**Figure 3:**
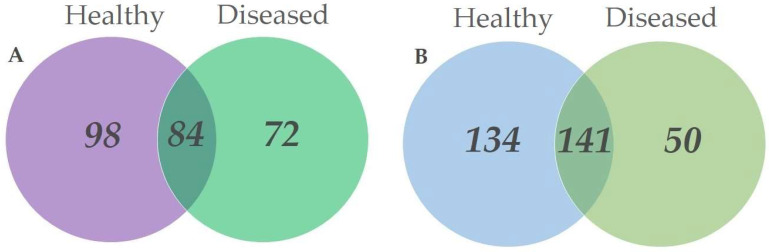
Venn diagrams showing the diversity and overlap of fungal taxa in different substrates collected in *Ulmus glabra* stands. (**A**) roots from healthy-looking and diseased trees; (**B**) leaves from healthy-looking and diseased trees.

Among all fungal taxa in root samples, 98 (38.6%) were exclusively found in the roots of healthy trees, 72 (28.3%)—in the roots of diseased trees, and 84 (33.1%) were in both sample types Figure 3A. Among all fungal taxa in leaf samples, 134 (41.2%) were exclusively found in leaves of healthy trees, 50 (15.4%)—in leaves of diseased trees, and 141 (43.4%) were in both sample types Figure 3B.

In root samples, the most abundant fungal taxa were *Trichocladium griseum* (32.9%), *Penicillium restrictum* (21.2%), and Unidentified sp. 5238_7 (12.6%). The relative abundance of *T. griseum* and *P. restrictum* was significantly higher in the roots of diseased trees than in the roots of healthy-looking ones (*p* < 0.05). Unidentified sp. 5238_20 (4.56%), *Absidia glauca* (0.36%), and *Solicoccozyma aeria* (0.29%) were found only in the roots of diseased trees. By contrast, Unidentified sp. 5238_7 (12.6%), Unidentified sp. 5238_78 (0.8%), *Xerocomellus porosporus* (0.66%), and *Thyrostroma tiliae* (0.58%) were only found in roots of healthy-looking trees, and only in the site G1 Table 1 and Table 3.

The most common fungal taxa in leaves were *Trichomerium* sp. 5238_8 (12.30%), *Aureobasidium pullulans* (12.03%), *Cladosporium* sp. 5238_5 (11.73%), and *Vishniacozyma carnescens* (9.86%). Among the 30 most common fungal taxa, only *Capnodium* sp. 5238_75 showed a significantly higher relative abundance in leaves of diseased trees than in leaves of healthy-looking trees (5.6% vs. 0.04%), while *Lapidomyces aloidendircola* was found exclusively in leaves of healthy-looking trees (0.48%) Table 4.

**Figure 4:**
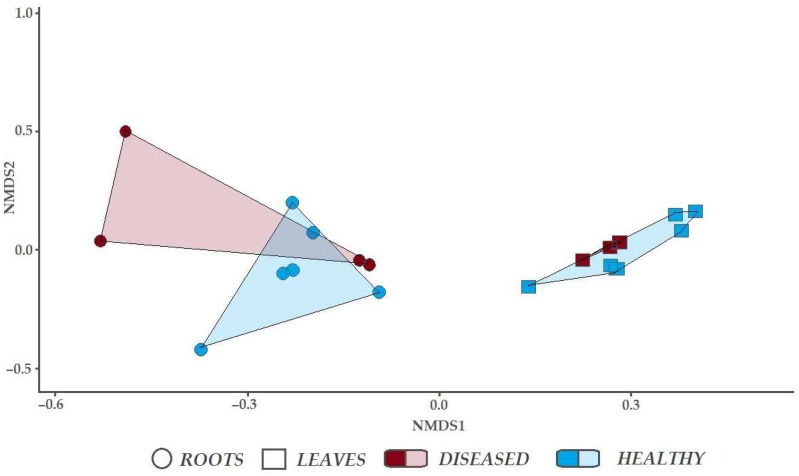
Non-metric multidimensional scaling for fungal communities associated with leaves and roots of healthy-looking and diseased *Ulmus glabra*.

NMDS revealed that fungal communities in the roots of healthy-looking and diseased trees were only partly overlapping Figure 4. However, analysis of similarities (ANOSIM) showed that the difference was insignificant (R = −0.1481, *p* > 0.05). Fungal communities in the leaves of healthy-looking and diseased trees were completely overlapping (R = −0.06349, *p* > 0.05).

## 4. Discussion

Climate change is predicted to have a major effect on tree health [36,37,38]. This includes the effects of global warming on the emergence of new pest insects, and pathogenic fungi. Such damages by pests and/or pathogens may cause physiological disturbances in trees and may also predispose to attacks by other pests or pathogens [37,39]. DED represents an example of a complex disease where both bark beetles and pathogenic fungi are involved, thereby leading to devastating consequences for elm trees and associated biodiversity.

Indeed, the results of our study provided evidence that DED has an effect on fungal diversity associated with the leaves and roots of *U. glabra*. Although this is not surprising, it could have been expected that the leaves and roots of diseased trees would have a higher diversity of fungi than healthy trees due to the loss of resistance and new niche opportunities. The decrease in fungal diversity was in both roots and leaves of diseased trees. Figure 3 shows that this was a common response in different tissues. Although the decrease in diversity of fungal taxa was due to the DED infection, a larger overall number of fungal taxa, both in healthy-looking and diseased tree leaves and roots, could be recovered using deeper sequencing Figure 2. Nevertheless, the results showed a relatively high diversity of fungal taxa in the leaves and roots of *U. glabra*, which is comparable to other tree species [40,41].

It was shown before that the species abundance and diversity in different plant tissues are affected by various biotic and abiotic factors. Abiotic factors such as soil water availability [42], soil acidity and nutrient availability [43,44], and litter characteristics [45] may strongly influence fungal communities in the soil and tree roots. The results of the present study demonstrate that due to the biotic factor, namely DED, the diversity of fungal taxa in roots in different study sites was reduced by up to 20%, which is a significant reduction. Anyway, in roots, the Shannon diversity index was relatively low and differed substantially between healthy-looking and diseased trees Table 2.

After the DED infection, leaves start to wilt, and their vitality rapidly deteriorates [11,20]. Although the age of leaves may also have an effect on fungal diversity [46,47], leaves in the present study were of similar age and collected in the same stands, showing that observed changes were largely due to the DED. The loss of fungal diversity in leaves of diseased trees was even more pronounced than in roots, as in different stands, it was up to 31%. However, differently from roots, in leaves, the Shannon diversity index was moderate and similar between healthy-looking and diseased trees Table 2.

In addition to changes in fungal diversity, there were also changes in the relative abundance of fungal taxa. In roots, several dominant fungal taxa had a significantly higher relative abundance in healthy trees than in diseased ones. Among these, there were *Roesleria subterranea* (11.45% vs. 0.03%), *Cyclocybe* sp. 5238_22 (9.01% vs. 0.04%), and Unidentified sp. 5238_39 (4.91% vs. 0.47%). Several common fungal taxa, such as Unidentified sp. 5238_7 (37.88%), Unidentified sp. 5238_78 (2.39%), *Xerocomellus porosporus* (1.98%), were not detected in roots of diseased trees Table 3. Among all fungal taxa, Unidentified sp. 5238_7 was the most abundant in roots of healthy trees, suggesting that it can be functionally important. The latter also shows that taxonomic species identification is still limited using available sequence databases such as Genbank and that important species can remain unidentified [48], even though these can be of special interest [49]. Overall, in the roots of healthy-looking trees, only 27 fungal taxa could be reliably identified at the species or genus level. *Roesleria subterranea* was the second most common fungal taxon in the roots of healthy-looking trees. It is commonly found in the roots of various deciduous trees and can grow saprotrophically on dead wood, but it can also cause root rot in living plants such as grape vines [50]. It appears that in the roots of diseased elm trees, *R. subterranea* is either rapidly outcompeted by other fungi or that its preferred habitat is living roots.

In the roots of diseased trees, the first three most common fungal taxa accounted for 86.9% of all fungal sequences Table 3. Among these was *T. griseum*, which is commonly detected in the soil and in association with plant leaves, but the exact functional role is obscure. However, its predominant occurrence in the roots of diseased trees may suggest that it can be an early colonizer of dying or dead plant tissues. The second most common fungus was *P. restrictum*, which has worldwide distribution and is generally considered a typical soil fungus. Nevertheless, it was reported from different natural environments, including different plant tissues and air or water samples [51,52]. *Penicillium restrictum* was shown to antagonize plant and soil-borne pathogens such as *Rhizoctonia solani*, *Botrytis cinerea*, *Phoma exigua*, and many *Fusarium* species (*F. avenaceum*, *F. culmorum*, *F. equiseti*, *F. oxysporum* and *F. solani*) [53]. Similarly to *T. griseum*, in the present study, it can be an early colonizer of dying roots.

Differently from roots, the most common fungal taxa in leaves of healthy-looking and diseased trees were largely the same Table 4. Among these was *A. pullulans*, which is a widespread endophyte or epiphyte of plants [54]. Its common occurrence in leaves of both healthy-looking and diseased trees suggests that it may occupy a broad ecological niche. *Vishniacozyma carnescens* is endophytic yeast adapted to a cold climate. *Vishniacozyma* sp. was shown to have adverse effects on species such as *Cladosporium uredinicola*, *Coprinellus micaceus*, or *Alternaria* sp. *Vishniacozyma* may also be effective in the biocontrol of pre- and post-harvest infections in horticulture [55,56].

In summary, the results demonstrated that functional tissues of elm trees were inhabited by a high richness of fungal taxa, the diversity of which rapidly declined following the tree infection by DED fungi. Despite the diversity decline in DED-infected trees, the relative abundance of fungal taxa and community compositions remained largely unchanged, suggesting that rare fungal taxa, which may depend on living plant tissues such as biotrophs and/or endophytes, were mostly affected. Consequently, DED not only devastates elm trees but also greatly affects the associated biodiversity.

## Figures and Tables

**Table 1:** Characteristics of sample sites where roots and leaves from healthy-looking and diseased trees were sampled.

Site *	Vegetation Type ^a^	Forest Site Type ^b^	Stand Age	Tree Species Composition in Decreasing Order of Occurrence	Location
G1	Šcs	ox	90	*Pinus sylvestris*, *Picea abies*, *Quercus robur*	54.872557° N, 023.97829° E
G2	Šcl	ox	40	*Alnus incana*, *Populus tremula*, *Acer platanoides*, *Tilia cordata*, *Ulmus glabra*	54.923794° N, 023.762145° E
G3	Lcl	mox	50	*Alnus incana*, *Alnus glutinosa, Acer platanoides*, *Ulmus glabra*, *Betula pendula*	55.010736° N, 024.529356° E
G4	Ncl	ox	50	*Alnus incana*, *Ulmus glabra*, *Tilia cordata*, *Populus tremula*, *Pinus sylvestris*	54.598671° N, 025.23843° E
G5	Šds	hox	100	*Quercus robur*, *Picea abies*, *Tilia cordata*, *Fraxinus excelsior*, *Ulmus glabra*	54.938315° N, 023.760325° E
G6	Šdp	hox	50	*Alnus incana*, *Betula pendula*, *Fraxinus excelsior*, *Ulmus glabra*, *Acer platanoides*	55.27918° N, 024.950571° E

* The site number is as in Figure 1. ^a^ N: normal humidity; L: temporarily waterlogged soils; Š: normal humidity slopes (>15°). c: moderate fertility; d: high fertility. l: light soil texture; s: heavy soils; p: binary soils [27], ^b^ ox: *oxalidosum*; mox: *myrtillo-oxalidosa*; hox: *Hepatico-oxalidosa* [28].

**Table 2:** Generated high-quality fungal sequences and detected a diversity of fungal taxa in different substrates from six *Ulmus glabra* sampling sites in Lithuania.

Site No.	Tree Status *	Roots			Leaves		
		Sequences	Taxa	Shannon Index (H)	Sequences	Taxa	Shannon Index (H)
G1	H	3962	77	1.82	652	76	3.07
	D	-	-	-	-	-	-
G2	H	454	64	2.81	577	61	2.83
	D	7443	46	0.95	-	-	-
G3	H	783	37	0.94	3444	140	3.06
	D	3853	54	0.32	1105	99	3.2
G4	H	270	40	2.9	2730	142	3.1
	D	381	70	3.42	873	100	3.09
G5	H	342	41	2.54	210	46	2.93
	D	-	-	-	-	-	-
G6	H	161	25	1.09	4059	146	2.89
	D	305	60	3.1	1095	101	3.1
TOTAL	H	5972	182	2.81	11,672	275	3.34
	D	11,982	156	1.66	3073	191	3.46
ALL		17,954	254	2.58	14,745	325	3.41

* H–healthy, D–diseased.

**Table 3:** Occurrence and relative abundance of the 30 most common fungal taxa (shown as a proportion of all high-quality fungal sequences) in roots of healthy-looking (H) and diseased (D) trees of *Ulmus glabra* sampled from different sites (G1–G6) in Lithuania.

Phylum *	Species Name	Reference Number	Similarity, %	G1		G2		G3		G4		G5		G6		Total		
				H, %	D, %	H, %	D, %	H, %	D, %	H, %	D, %	H, %	D, %	H, %	D, %	H, %	D, %	All, %
A	*Trichocladium griseum*	MN643061	100	0.03	-	12.56	78.34	0.64	0.08	-	0.79	0.29	-	0.62	1.31	1.09	48.75	32.90
A	*Penicillium restrictum*	MT090009	100	0.05	-	1.10	0.59	2.30	95.80	3.33	4.46	4.09	-	0.62	1.97	0.82	31.36	21.20
B	Unidentified sp. 5238_7	KC588795	99	57.09	-	-	-	-	-	-	-	-	-	-	-	37.88	-	12.60
A	Unidentified sp. 5238_20	KX193283	100	-	-	-	11.00	-	-	-	-	-	-	-	-	-	6.84	4.56
A	*Roesleria subterranea*	KU666540	100	-	-	-	-	84.04	0.10	9.63	-	-	-	-	-	11.45	0.03	3.83
B	*Cyclocybe* sp. 5238_22	MW644549	97	13.55	-	-	-	-	-	-	-	-	-	0.62	1.64	9.01	0.04	3.02
A	Unidentified sp. 5238_39	JF519577	100	5.78	-	13.88	0.03	0.13	0.21	-	0.79	-	-	-	14.10	4.91	0.47	1.94
B	*Trechisporales* sp. 5238_32	KU973892	100	1.59	-	27.31	-	0.26	-	-	9.97	-	-	0.62	22.95	3.18	0.90	1.66
A	*Leptodontidium camptobactrum*	MH857172	100	0.03	-	-	2.42	-	-	-	-	-	-	-	-	0.02	1.50	1.01
B	*Mycena leptocephala*	MT644911	100	0.03	-	-	-	-	0.31	-	0.79	-	-	80.12	-	2.18	0.13	0.81
A	Unidentified sp. 5238_78	MH636731	99	3.61	-	-	-	-	-	-	-	-	-	-	-	2.39	-	0.80
B	*Malassezia restricta*	MK336446	99	0.20	-	3.96	0.01	0.64	0.21	8.15	15.49	0.88	-	-	4.92	0.94	0.69	0.77
B	*Xerocomellus porosporus*	HM190086	99	2.98	-	-	-	-	-	-	-	-	-	-	-	1.98	-	0.66
M	*Podila minutissima*	MT366014	100	0.15	-	0.22	1.42	-	-	0.37	-	0.29	-	-	0.33	0.15	0.89	0.65
B	*Thyrostroma tiliae*	MK751738	100	2.62	-	-	-	-	-	-	-	-	-	-	-	1.74	-	0.58
A	*Dothideomycetes* sp. 5238_97	LR864332	99	-	-	6.83	0.01	-	0.44	2.96	8.66	1.46	-	-	-	0.74	0.43	0.53
A	*Camposporium multiseptatum*	NR171863	100	2.32	-	-	-	-	-	-	-	0.29	-	0.62	0.33	1.57	0.01	0.53
A	*Trichoderma atroviride*	MT514373	99	0.03	-	-	1.10	-	-	-	-	0.29	-	-	0.66	0.03	0.70	0.48
B	*Entoloma strigosissimum*	JF908004	99	-	-	7.27	-	-	0.18	10.00	4.72	-	-	-	-	1.00	0.21	0.47
B	*Conocybe nigrescens*	MK217423	100	-	-	-	-	-	-	-	-	22.51	-	-	-	1.29	-	0.43
A	*Cadophora orchidicola*	MT436755	100	0.20	-	-	0.13	0.89	0.10	-	1.57	3.22	-	3.11	6.89	0.52	0.34	0.40
A	*Cladosporium* sp. 5238_5	MT645945	100	0.13	-	1.32	0.01	1.02	-	6.67	5.77	0.29	-	-	3.28	0.64	0.28	0.40
B	*Infundibulicybe geotropa*	KT122792	100	-	-	-	-	-	-	-	-	20.76	-	-	-	1.19	-	0.40
Z	*Absidia glauca*	EU484257	100	-	-	-	0.87	-	-	-	-	-	-	-	-	-	0.54	0.36
A	Unidentified sp. 5238_132	KX222675	100	1.34	-	-	-	-	0.03	-	-	-	-	0.62	2.30	0.90	0.07	0.35
B	Unidentified sp. 5238_124	LR874952	100	-	-	-	-	-	0.03	-	-	16.96	-	-	-	0.97	0.01	0.33
B	*Solicoccozyma terricola*	MH487580	100	0.05	-	-	0.74	0.13	-	-	-	-	-	-	-	0.05	0.46	0.32
A	Unidentified sp. 5238_145	MK627256	99	1.26	-	-	-	-	0.05	-	-	-	-	-	1.64	0.84	0.06	0.32
A	*Pseudogymnoascus* sp. 5238_46	MT367251	100	-	-	0.22	0.63	0.13	0.05	-	0.26	0.29	-	0.62	0.66	0.07	0.43	0.31
B	*Solicoccozyma aeria*	MT596205	100	-	-	-	0.70	-	-	-	-	-	-	-	-	-	0.43	0.29
				93.03	0.00	74.67	98.02	90.17	97.59	41.11	53.28	71.64	0.00	87.58	62.95	87.54	95.57	92.90

* A—Ascomycota, B—Basidiomycota, Z—Zygomycota, M—Mucoromycota.

**Table 4:** Occurrence and relative abundance of the 30 most common fungal taxa (shown as a proportion of all high-quality fungal sequences) in leaves of healthy-looking (H) and diseased (D) trees of *Ulmus glabra* sampled from different sites (G1–G6) in Lithuania.

Phylum *	Species Name	Reference Number	Similarity, %	G1		G2		G3		G4		G5		G6		Total		
				H, %	D, %	H, %	D, %	H, %	D, %	H, %	D, %	H, %	D, %	H, %	D, %	H, %	D, %	All, %
A	*Trichomerium* sp. 5238_8	MT223865	94	5.98	-	14.56	-	10.54	10.23	20.22	8.59	-	-	9.76	13.52	12.66	10.93	12.30
A	*Aureobasidium pullulans*	MT645930	100	3.68	-	10.92	-	9.93	13.12	7.33	4.93	5.24	-	17.10	15.98	12.09	11.81	12.03
A	*Cladosporium* sp. 5238_5	MT645945	100	5.83	-	11.44	-	5.72	7.87	7.69	13.29	27.62	-	18.34	11.87	11.96	10.84	11.73
B	*Vishniacozyma carnescens*	MT595884	100	15.03	-	11.79	-	10.31	7.69	5.64	9.62	13.33	-	10.31	10.68	10.01	9.31	9.86
A	Unidentified sp. 5238_14	KX147989	90	-	-	-	-	24.16	18.55	2.93	0.11	-	-	-	-	7.81	6.70	7.58
A	Unidentified sp. 5238_9	MT236420	100	2.76	-	0.69	-	2.64	0.54	8.75	9.51	-	-	3.46	6.39	4.35	5.17	4.52
A	*Coniozyma* sp. 5238_17	MW764574	100	8.13	-	0.35	-	3.02	1.54	13.08	1.37	0.48	-	0.51	4.20	4.63	2.44	4.17
B	Unidentified sp. 5238_30	MN903728	100	13.50	-	5.55	-	4.33	4.98	1.61	2.06	6.19	-	1.84	2.28	3.50	3.19	3.44
A	*Didymella* sp. 5238_12	MT453298	100	2.30	-	8.32	-	1.25	0.54	4.58	2.52	4.76	-	2.84	5.11	3.16	2.73	3.07
B	Unidentified sp. 5238_33	KU057810	92	3.37	-	0.52	-	0.87	1.81	1.06	2.29	1.90	-	4.70	5.30	2.57	3.19	2.70
B	*Buckleyzyma aurantiaca*	KX096691	100	3.37	-	0.52	-	1.54	0.54	2.75	1.15	0.48	-	1.53	1.55	1.91	1.07	1.74
A	*Lemonniera* sp. 5238_6	KX096679	99	14.42	-	2.43	-	1.10	1.00	1.47	0.80	0.95	-	0.42	0.27	1.77	0.68	1.55
A	Unidentified sp. 5238_53	AB476498	97	1.38	-	-	-	1.22	1.63	0.66	-	-	-	2.64	1.10	1.61	0.98	1.48
B	*Filobasidium wieringae*	MN128850	100	0.92	-	0.87	-	1.39	2.44	2.42	0.92	0.48	-	0.60	0.27	1.31	1.24	1.30
A	*Capnodium* sp. 5238_75	AJ244240	96	-	-	-	-	0.03	-	0.15	19.70	-	-	-	-	0.04	5.60	1.20
A	*Ramularia vizellae*	MK012421	100	0.46	-	2.25	-	1.74	3.35	0.44	0.69	1.43	-	0.69	0.18	1.05	1.46	1.13
B	*Tremellales* sp. 5238_52	MG827438	100	0.61	-	0.52	-	0.44	0.72	1.98	1.03	2.38	-	0.49	0.73	0.88	0.81	0.87
B	*Rhodosporidiobolus colostri*	MT502792	100	0.31	-	0.52	-	2.15	1.81	0.11	-	-	-	0.20	1.00	0.78	1.01	0.83
B	*Filobasidium dingjieense*	MK050343	100	-	-	1.21	-	2.61	0.81	-	-	-	-	0.02	0.37	0.84	0.42	0.75
B	*Filobasidium mucilaginum*	MK050349	99	-	-	0.17	-	0.12	0.27	1.06	1.72	0.48	-	0.86	0.64	0.63	0.81	0.67
B	*Bannozyma yamatoana*	AF444634	99	-	-	-	-	0.61	1.63	0.70	-	-	-	0.73	0.37	0.63	0.72	0.64
B	*Sporobolomyces roseus*	MT502791	100	0.15	-	0.35	-	0.06	0.36	0.15	0.11	4.76	-	1.29	1.10	0.66	0.55	0.64
A	Unidentified sp. 5238_113	KT328793	90	1.53	-	14.21	-	-	-	-	-	-	-	0.02	0.09	0.80	0.03	0.64
B	*Dioszegia* sp. 5238_18	LT548261	100	1.38	-	1.56	-	0.38	0.27	0.26	0.23	0.95	-	0.69	1.00	0.61	0.52	0.59
B	*Malassezia restricta*	MK336446	99	0.15	-	-	-	0.17	0.54	0.22	3.09	4.76	-	0.07	2.19	0.22	1.85	0.56
A	*Neosetophoma* sp. 5238_42	MN244543	100	0.31	-	0.35	-	0.29	0.18	0.18	1.72	0.48	-	0.64	0.64	0.42	0.78	0.50
B	*Curvibasidium cygneicollum*	KY102972	100	-	-	0.17	-	0.90	1.18	0.04	-	-	-	0.22	0.46	0.37	0.59	0.41
A	*Lapidomyces aloidendricola*	NR173048	98	-	-	-	-	0.03	-	2.01	-	-	-	-	-	0.48	-	0.38
B	*Cryptococcus* sp. 5238_131	KT314197	99	0.15	-	1.04	-	0.32	0.09	0.22	0.80	0.95	-	0.40	0.37	0.38	0.39	0.38
A	*Chaetothyriales* sp. 5238_142	KX402745	99	-	-	-	-	0.75	1.09	0.18	0.92	-	-	-	-	0.27	0.65	0.35
			All	85.74	0.00	90.29	0.00	88.62	84.80	87.88	87.17	77.62	0.00	80.37	87.67	88.40	86.50	88.00

* A—Ascomycota, B—Basidiomycota.

## Data Availability

The data is available upon request from the corresponding author.

## Supplementary Materials

The following supporting information can be downloaded at: https://www.mdpi.com/article/10.3390/microorganisms10112228/s1, Table S1: Occurrence and relative abundance of fungal taxa (shown as a proportion of all high-quality fungal sequences) in roots of healthy-looking (H) and diseased (D) trees of Ulmus glabra sampled from different sites (G1–G6) in Lithuania; Table S2: Occurrence and relative abundance of fungal taxa (shown as a proportion of all high-quality fungal sequences) in leaves of healthy-looking (H) and diseased (D) trees of Ulmus glabra sampled from different sites (G1–G6) in Lithuania.

## References

[1] Jürisoo L., Adamson K., Padari A., Drenkhan R. (2019). Health of elms and Dutch elm disease in Estonia. Eur. J. Plant Pathol..

[2] Motiejūnaitė J., Kutorga E., Kasparavičius J., Lygis V., Norkutė G. (2016). New records from Lithuania of fungi alien to Europe. Mycotaxon.

[3] Thor G., Johansson P., Jönsson M.T. (2010). Lichen diversity and red-listed lichen species relationships with tree species and diameter in wooded meadows. Biodivers. Conserv..

[4] Richens R.H. (1983). Elm.

[5] Heybroek H.M., Sticklen M.B., Sherald J.L. (1993). The Dutch Elm Breeding Program. Dutch Elm Disease Research.

[6] Jüriado I., Liira J., Paal J., Suija A. (2009). Tree and stand level variables influencing diversity of lichens on temperate broad-leaved trees in boreo-nemoral floodplain forests. Biodivers. Conserv..

[7] Corfixen P., Parmasto E. (2005). *Hymenochaete ulmicola*, sp.nov. (Hymenochaetales). Mycotaxon.

[8] Kalamees K., Hausknecht A., Vauras J. (2013). Checklist of the genera *Conocybe* and *Pholiotina* (Agaricales, Agaricomycetes) in Estonia. Folia Cryptogam. Est..

[9] Brasier C.M. (1991). *Ophiostoma novo-ulmi* sp. nov., causative agent of current Dutch elm disease pandemics. Mycopathologia.

[10] Brasier C.M., Dunne C.P. (2000). Intercontinental spread and continuing evolution of the Dutch elm disease pathogens. The Elms: Breeding, Conservation and Disease Management.

[11] Yadeta K.A., Thomma B.P.J. (2013). The xylem as battleground for plant hosts and vascular wilt pathogens. Front. Plant Sci..

[12] Gibbs J.N., Brasier C.M. (1973). Correlation between cultural characters and pathogenicity in *Ceratocystis ulmi* from Britain, Europe and America. Nature.

[13] Paoletti M., Buck K.W., Brasier C.M. (2005). Cloning and sequence analysis of the MA TB (MAT-2) genes from the three Dutch elm disease pathogens, *Ophiostoma ulmi*, *O. novo-ulmi* and *O. himal-ulmi*. Mycol. Res..

[14] Miyamoto T., Masuya H., Koizumi A., Yamaguchi T., Ishihara M., Yamaoka Y., Ohara M. (2019). A report of dieback and mortality of elm trees suspected of Dutch elm disease in Hokkaido, Japan. J. For. Res..

[15] Brasier C.M. (1983). A cytoplasmically transmitted disease of *Ceratocystis ulmi*. Nature.

[16] Fransen J.J. (1939). Elm Disease, Elm Beetles and Their Control. PhD Thesis.

[17] Webber J.F. (2004). Experimental studies on factors influencing the transmission of Dutch elm disease. For. Syst..

[18] Li Y., Wang Y., Xue H., Pritchard H.W., Wang X. (2017). Changes in the mitochondrial protein profile due to ROS eruption during ageing of elm (*Ulmus pumila* L.) seeds. Plant Physiol. Biochem..

[19] Venturas M., Nanos N., Gil L. (2014). The reproductive ecology of *Ulmus laevis* Pallas in a transformed habitat. For. Ecol. Manag..

[20] Santini A., Faccoli M. (2015). Dutch elm disease and elm bark beetles: A century of association. iFor.-Biogeosci. For..

[21] Anderbrant O., Yuvaraj J.K., Martin J.A., Gil L., Witzell J. (2017). Feeding by *Scolytus* bark beetles to test for differently susceptible elm varieties. J. Appl. Entomol..

[22] Liu L., Yang C., Xu X., Wang X., Liu M., Chen R., Tan F., Liu Y., Lin T., Liu Y. (2022). Unlocking the Changes of Phyllosphere Fungal Communities of Fishscale Bamboo (Phyllostachys heteroclada) under Rhombic-Spot Disease Stressed Conditions. Forests.

[23] Martín J.A., Witzell J., Blumenstein K., Rozpedowska E., Helander M., Sieber T.N., Gil L. (2013). Resistance to Dutch elm disease reduces presence of xylem endophytic fungi in elms (*Ulmus* spp.). PLoS ONE.

[24] Witzell J., Martín J.A., Blumenstein K., Verma V.C., Gange A.C. (2014). Ecological aspects of endophyte-based biocontrol of forest diseases. Advances in Endophytic Research.

[25] Busby P.E., Ridout M., Newcombe G. (2016). Fungal endophytes: Modifiers of plant disease. Plant. Mol. Biol..

[26] Terhonen E., Blumenstein K., Kovalchuk A., Asiegbu F.O. (2019). Forest tree microbiomes and associated fungal endophytes: Functional roles and impact on forest health. Forests.

[27] Vaičys M. (2001). Miško dirvožemių klasifikacija. Lietuvos Dirvožemiai.

[28] Karazija S. (1988). Lietuvos Miško Tipai.

[29] Marčiulynas A., Marčiulynienė D., Mishcherikova V., Franić I., Lynikienė J., Gedminas A., Menkis A. (2022). High Variability of Fungal Communities Associated with the Functional Tissues and Rhizosphere Soil of *Picea abies* in the Southern Baltics. Forests.

[30] Ihrmark K., Bodeker I.T.M., Cruz-Martinez K., Friberg H., Kubartova A., Schenck J., Strid Y., Stenlid J., Brandstrom-Durling M., Clemmensen K.E. (2012). New primers to amplify the fungal ITS2—Evaluation by 454-sequencing of artificial and natural communities. FEMS Microbiol. Ecol..

[31] White T.J., Bruns T., Lee S., Taylor J., Innis M.A., Gelfand D.H., Sninsky J.J., White T.J. (1990). Amplification and direct sequencing of fungal ribosomal RNA genes for phylogenetics. PCR Protocols: A Guide to Methods and Applications.

[32] Shannon C.E. (1948). A mathematical theory of communication. Bell Syst. Tech. J..

[33] Magurran A.E. (1988). Ecological Diversity and Its Measurement.

[34] Oksanen J., Blanchet F.G., Kindt R., Legendre P., Minchin P.R., O’Hara R.B., Simpson G.L., Solymos P., Stevens M.H.H., Wagner H. (2013). Community Ecology Package. R Package Version 2. https://cran.r-project.org/web/packages/vegan/index.html.

[35] R Core Team (2021). R: A Language and Environment for Statistical Computing.

[36] Sieber T.N. (2007). Endophytic fungi in forest trees: Are they mutualists?. Fungal Biol. Rev..

[37] Gonthier P., Gennaro M., Nicolotti G. (2006). Effects of water stress on the endophytic mycota of *Quercus robur*. Fungal Diver..

[38] Speer J.H., Grissino-Mayer H.D., Orvis K.H., Greenberg C.H. (2009). Climate response of five oak species in the eastern deciduous forest of the southern Appalachian Mountains, USA. Can. J. For. Res..

[39] Pureswaran D.S., Roques A., Battisti A. (2018). Forest insects and climate change. Curr. For. Rep..

[40] Nguyen M.H., Shin K.C., Lee J.K. (2021). Fungal Community Analyses of Endophytic Fungi from Two Oak Species, *Quercus mongolica* and *Quercus serrata*, in Korea. Mycobiology.

[41] Agan A., Drenkhan R., Adamson K., Tedersoo L., Solheim H., Børja I., Matsiakh I., Timmermann V., Nagy N.E., Hietala A.M. (2020). The relationship between fungal diversity and invasibility of a foliar niche—The case of ash dieback. J. Fungus.

[42] Barbier S., Balandier P., Gosselin F. (2009). Influence of several tree traits on rainfall partitioning in temperate and boreal forests: A review. Ann. For. Sci..

[43] Carnol M., Bazgir M. (2013). Nutrient return to the forest floor through litter and throughfall under 7 forest species after conversion from Norway spruce. For. Ecol. Manag..

[44] Augusto L., Achat D.L., Bakker M.R., Bernier F., Bert D., Danjon F., Khlifa R., Meredieu C., Trichet P. (2015). Biomass and nutrients in tree root systems—Sustainable harvesting of an intensively managed *Pinus pinaster* (Ait.) planted forest. GCB Bioenergy.

[45] Hobbie S.E., Reich P.B., Oleksyn J., Ogdahl M., Zytkowiak R., Hale C., Karolewski P. (2006). Tree species effects on decomposition and forest floor dynamics in a common garden. Ecology.

[46] Petrini O., Stone J., Carroll F.E. (1982). Endophytic fungi in evergreen shrubs in western Oregon: A preliminary study. Can. J. Bot..

[47] Arnold A.E., Herre E.A. (2003). Canopy cover and leaf age affect colonization by tropical fungal endophytes: Ecological pattern and process in *Theobroma cacao* (Malvaceae). Mycologia.

[48] Menkis A., Marčiulynas A., Gedminas A., Lynikienė J., Povilaitienė A. (2015). High-throughput sequencing reveals drastic changes in fungal communities in the phyllosphere of Norway spruce (*Picea abies*) following invasion of the spruce bud scale (*Physokermes piceae*). Microb. Ecol..

[49] Leray M., Knowlton N., Ho S.L., Nguyen B.N., Machida R.J. (2019). GenBank is a reliable resource for 21st century biodiversity research. Proc. Natl. Acad. Sci. USA.

[50] Neuhauser S., Huber L., Kirchmair M. (2011). Is *Roesleria subterranea* a primary pathogen or a minor parasite of grapevines? Risk assessment and a diagnostic decision scheme. Eur. J. Plant Pathol..

[51] Kowalik M., Muras P. (2007). Fungi Occurring on the Fallen Leaves of Rhododendron.

[52] Slinkina N.N., Pivkin M.V., Polokhin O.V. (2010). Filamentous fungi of the submarine soils of the Sakhalin Gulf (Sea of Okhotsk). Russ. J. Mar. Biol..

[53] Nicoletti R., de Stefano M. (2012). *Penicillium restrictum* as an antagonist of plant pathogenic fungi. Dyn. Biochem. Process. Biotechnol. Mol. Biol..

[54] Andrews J.H. (2006). Population Growth and the Landscape Ecology. Microbial Ecology of Aerial Plant Surfaces.

[55] Gramisci B.R., Lutz M.C., Lopes C.A., Sangorrín M.P. (2018). Enhancing the efficacy of yeast biocontrol agents against postharvest pathogens through nutrient profiling and the use of other additives. Biol. Cont..

[56] Pawlikowska E., James S.A., Breierova E., Antolak H., Kregiel D. (2019). Biocontrol capability of local *Metschnikowia* sp. isolates. Antonie Van Leeuwenhoek.

